# Supplementary material for: Seminoma and Embryonal Carcinoma Footprints Identified by Analysis of Integrated Genome-Wide Epigenetic and Expression Profiles of Germ Cell Cancer Cell Lines
Source: PLoS One. 2014 Jun 2;9(6):e98330. doi: 10.1371/journal.pone.0098330 (PMC4041891; doi:10.1371/journal.pone.0098330)
Supplement: File S1 — ZIP file containing DMRforPairs output for significant regions. Please start from the html files. (ZIP) [file pone.0098330.s008.zip › figures/36732.pdf]

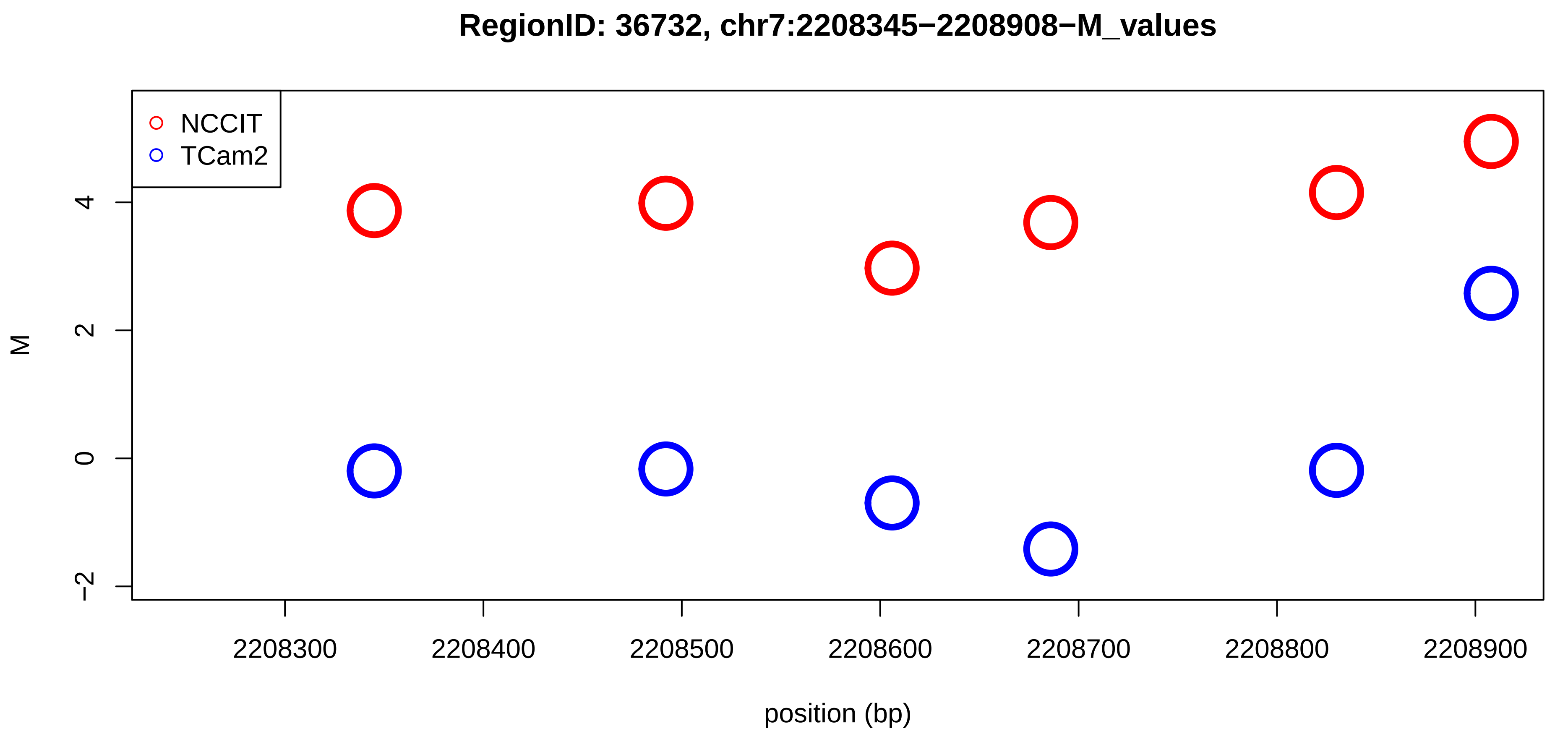
RegionID: 36732, chr7:2208345−2208908−M_values
NCCIT
TCam2
M
−2
0
2
4
2208300
2208400
2208500
2208600
2208700
2208800
2208900
position (bp)

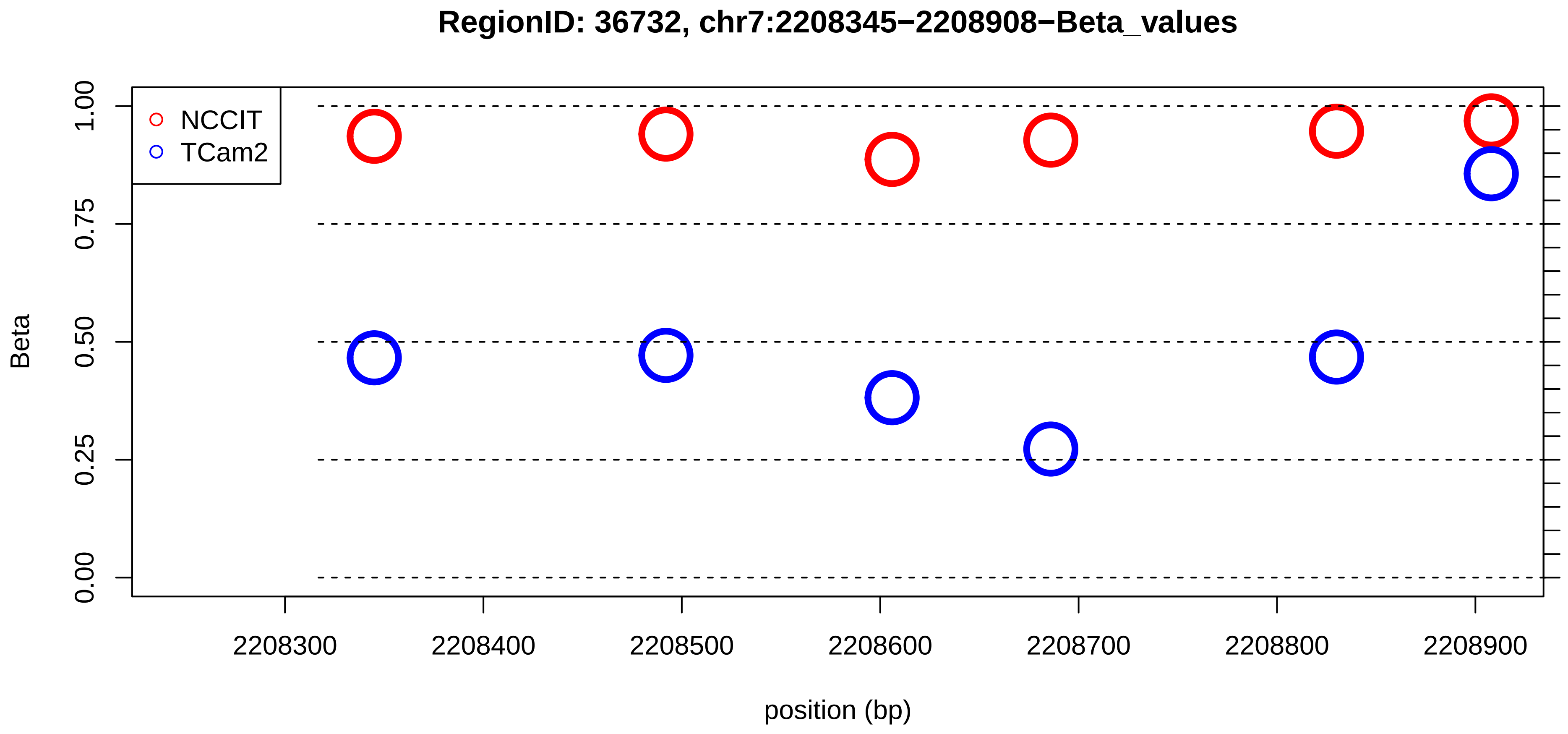
RegionID: 36732, chr7:2208345−2208908−Beta_values
NCCIT
TCam2
Beta
0.00
0.25
0.50
0.75
1.00
2208300
2208400
2208500
2208600
2208700
2208800
2208900
position (bp)
